# Postoperative effects of bilateral sphenopalatine ganglion blockade in septorhinoplasty operations; double-blind randomized clinical trial

**DOI:** 10.1016/j.bjorl.2023.101373

**Published:** 2023-12-02

**Authors:** Erhan Gökçek, Gunay Kozan

**Affiliations:** a Health Sciences University Diyarbakir Gazi Yasargil Research and Education Hospital, Department of Anaesthesiolgy and Reanimation, Diyarbakir, Turkey; b Dicle University, Faculty of Medicine, Department of Otorhinolaryngology, Diyarbakir, Turkey

**Keywords:** Septorhinoplasty surgery, Sphenopalatine ganglion blockade, Postoperative pain, Nausea and vomiting, Laryngospasm

## Abstract

**Objective:**

We aimed to investigate the effect of bilateral sphenopalatine ganglion blockade (SPGB) on the main postoperative complications in septorhinoplasty operations.

**Methods:**

In this randomized, controlled, prospective study, 80 cases planned for Septorhinoplasty operations under general anesthesia were included in the study. The cases were divided into two groups; SPGB was performed with 2 mL of 0.25% bupivacaine bilaterally 15 min before the end of the operation in the SPGB group (Group S, n = 40). In the control group (Group C, n = 40), 2 mL of 0.9% NaCl solution was applied into both SPG areas. In the recovery unit after the operation; the pain and analgesic needs of the patients at 0, 2, 6 and 24 h were evaluated.

**Results:**

There was no statistically significant difference between the groups in terms of hemodynamic parameters (ASA, MBP, HR) (*p* > 0.05) All VAS values were statistically lower in Group S than in Group C (*p* < 0.05). In Group S, the need for analgesic medication was found in 5 cases between 0–2 h, whereas in Group C, this rate was found in 17 cases, and it was statistically significant (*p* < 0.05).

**Conclusion:**

Bilateral SPGB application was determined to provide better analgesia in the early postoperative period compared to the control group, it was concluded that further studies are needed to say that there are significant effects on laryngospasm and nausea-vomiting.

**Level of evidence:**

2, degree of recommendation B.

## Highlights

• Sphenopalatine ganglion blockade was very effective in postoperative pain control after septorhinoplasty operations.
• Sphenopalatine ganglion blockade resulted in less analgesic drug consumption after septorhinoplasty operations.
• Sphenopalatine ganglion blockade was not effective in postoperative nausea and vomiting after septorhinoplasty operations.

## Introduction

The sphenopalatine ganglion (SPG) is one of the peripheral parasympathetic ganglia and associated with the general sensory fibers and internal carotid plexus that do not synapse in this ganglion.^1^ SPG blocks are used in head and neck chronic pain syndromes, eye diseases and in the treatment of asthma.^2^ It has been shown that SPG blockade provides postoperative analgesia and reduces the need for analgesics, and it has been claimed to be superior to general anesthesia in nasal surgery.^3^ After septorhinoplasty operations performed under general anesthesia; laryngospasm, nausea-vomiting, headache and sore throat are some of the common complications in the early recovery period. This is resulted from both tracheal intubation and the surgical field’s contact with the oropharyngeal cavity.

Despite the important developments in analgesia and surgical techniques, there is no consensus on the excessive use of drugs for pain control after septorhinoplasty operations. A large number of painkillers; nonsteroidal anti-inflammatory drugs (NSAIDs), multimodal or balanced analgesic protocols including non-opioid analgesic drugs such as gabapentin, opioids and paracetamol with or without intravenous dexamethasone, and various NSAIDs in combination with low doses of opioids have been previously studied for pain after nasal surgeries.^4^, ^5^, ^6^, ^7^, ^8^, ^9^ However, systemic use of these agents will cause potential side effects. We plan to reduce the need for these systemic analgesic drugs and get rid of their potential side effects and costs due to the sphenopalatine ganglion glockade (SPGB) to be used.

The primary aim of our study was to test the hypothesis that SPG blockade provides more analgesia in the first 24 h postoperatively than the control group in septorhinoplasty operations. In addition, the secondary aim of our study was to investigate the effects of SPG blockade on the ability to further reduce nausea-vomiting, sore throat, and laryngospasm.

## Methods

### Study design and participants

**Figure 1:**
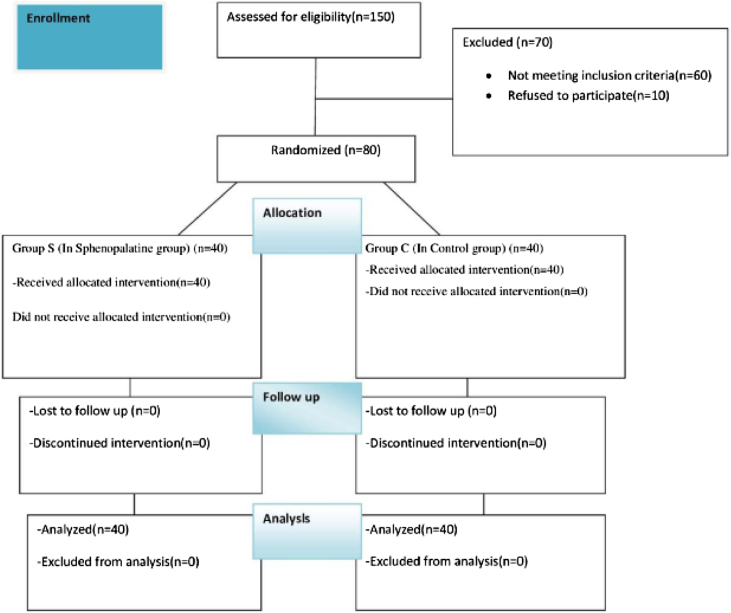
Recruitment and flow of patients.

Our study planned by randomized, double-blind and prospective study was conducted between May 2022 and May 2023. The approval for the research was granted by the Institutional Ethics Committee (decision nº: 66, 2022, Diyarbakir Gazi Yasargil Training and Research Hospital Ethics Committee). Written and spoken informed consent was obtained from all patients. Eighty patients aged 18–50 years, to be undergone septorhinoplasty under general anesthesia, were included in our study, after having been obtained the approval of the local ethics committee and informed consent form from the patients. Patients aged 18–50 who will undergo elective septorhinoplasty operations. Pregnant, known allergy to any of the study drugs, significant cardiac or renal pathology, regular sedative drug intake, atrioventricular block, myasthenia gravis, uncooperative (due to dementia, mental retardation, etc.), drug or alcohol addiction, and patients who did not want to participate in the study were excluded (Fig. 1).

### Random grouping

In Excel, a column of numbers 1–80 was established as column A. After numbering, column B of 80 corresponding random numbers was generated using a random function. Column A and column B were all selected and sorted in ascending order of column B, so that 80 numbers in column A were randomly arranged, with the first 40 random numbers assigned to the control group and the last 40 random numbers assigned to the intervention group. Then, the 80 randomly numbered cards were sequentially placed into 80 envelopes and the envelopes were ordered. After the patient agreed to participate in this study, the operator opened the envelopes sequentially according to the envelope number and obtained the random number and corresponding grouping. The patient did not know the grouping situation.

### Sample size calculation

In this parallel randomized controlled trial, the intervention group received bilateral sphenopalatine ganglion blockade, while the control group was left blank, and pain score was used as the primary outcome indicator. G-Power software (version 3.1.9.4; Kiel University, Kiel, Germany) was used to calculate the required sample size. According to previous literature.^10^ The sample size was based on a confidence level of 95% and power detection of 80%. Also, Standard Deviations (SDs) of VAS were 0.38 and 0.37, and the mean of difference (mean1 = 2.66 and mean2 = 4.16) was 1.5. Accordingly, the sample size was calculated at least 23 for each group based on a previous study. The minimum number of patients required was assumed to be 46 (23 in the experimental group and 23 in the control group), one-tailed alpha error 0.05, power 0.80, allocation ratio N1/N2 = 1, and effect size 0.75. A sample size of 80 was chosen to allow for incomplete data collection.

#### Preoperative ve peroperative procedure

Half an hour before the operation, all patients were premedicated with 0.03 mg/kg intramuscular midazolam (Dormicum®, Roche). In anesthesia induction, 2.5 mg/kg IV propofol (Pofol®), 1 μg/kg fentanyl (Fentanyl®, Janssen) bolus IV, 1 mg/kg IV arrhythmic (Aritmal®, Adeka) were administered. Muscle relaxation was enabled with IV 0.6 mg/kg rocuronium (Esmeron®, 10 mg/mL, Organon). After endotracheal intubation, 2% sevoflurane (Sevorane®, Abbot) was administered in an oxygen: air mixture to maintain anesthesia with an oxygen ratio of 40%. In addition to inhalation anesthesia, an IV infusion of 0.05–10 μg/kg/min remifentanil (Ultiva, Glaxo Welcome) was administered. The dose of remifentanil was increased or decreased when an increase or decrease of more than 20% of the baseline systolic arterial pressure was observed. Additional muscle relaxants were administered depending on the duration of the operation and the follow-up of neuromuscular blockade. When the heart rate fell below 50 beats/min, 0.5 mg of atropine was administered, and when the mean blood pressure (MBP) fell below 60 mmHg, it was administered with 10 mg of ephedrine.

Routine electrocardiography (ECG), non-invasive blood pressure and peripheral oxygen saturation (SpO_2_) monitoring were performed on the patients. Heart rate (HR), mean blood pressure (MBP), peripheral oxygen saturation was measured, and baseline values were recorded. Heart rate, MBP, SpO_2_ and minimum alveolar concentration (MAC) values were recorded during induction, intubation, at the 5th minute of anesthesia, every 15 min after and immediately after extubation. All measurements were made with Datex-Ohmeda anesthesia device equipment (AS/3, Datex®, Helsinki, Finland). The time from the induction of anesthesia to the patient’s admission to the recovery room was defined as the duration of anesthesia, and the time from the surgical incision to the closure of the skin was defined as the duration of surgery.

**Figure 2:**
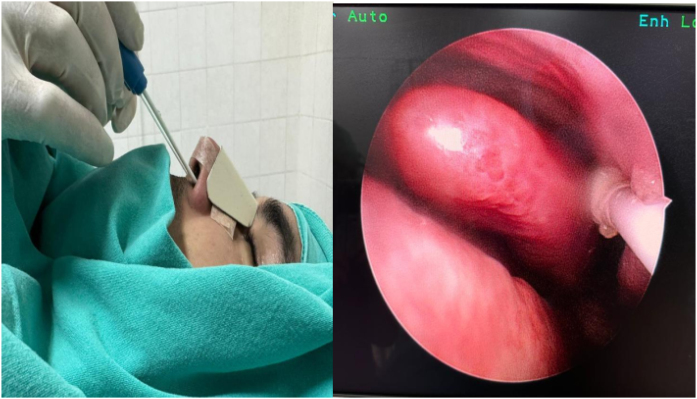
Sphenopalatine ganglion block application with the sphenocath device under general anesthesia.

The patients were randomized and divided into two groups as the sphenopalatine group (Group S, n = 40) and the control group (Group C, n = 40). It was applied to both groups by the same otolaryngologist with the SphenoCath Applicator (SphenoCath Applicator; Dolor Technologies, Scottsdale, Arizona) under the guidance of videoendoscopy. SPG blockade was performed by a surgeon blinded to the drug used. Patients in Group S were operated bilaterally with 2 mL of 0.025% adrenalin-free intranasal bupivacaine (Marcaine 0.5% vial, Astra-Zeneca, Germany) and 2 mL of 0.9% NaCl solution to each SPG site, 15 min before the end of the operation in the control group. The results were evaluated by an anesthesiologist who was not involved in the study (Fig. 2).

### Postoperative procedure

The extubated patients were taken to the post-anesthesia care unit (PACU) for 2 h for recovery evaluation. ECG, non-invasive blood pressure and SpO_2_ monitoring of the patients were performed. Afterwards, the patient was followed up in the otolaryngology service.

### Outcomes

#### Primary outcome

After the operation, pain in the recovery unit at 0th (t1), 2nd (t2), 6th (t3) and 24th (t4) hours was evaluated with the visual analog scale^11^ (0: no pain, 10: the most severe pain possible). In the follow-up of the patient, if VAS ≥ 5 points within the first 4 h after the operation, 1 mg/kg diclofenac sodium (Voltaren 75 mg amp, Novartis, Turkey) intramuscularly, if VAS ≥ 5 points within 5–24 h, diclofenac sodium (Voltaren Retard) 100 mg tb, Novartis, Turkey) 1 × 1 tb orally administration was planned.

#### Secondary outcomes

Postoperative analgesic requirement (0–2 h, 2–24 h ve total), presence of laryngospasm and nausea-vomiting scale in the first 24 h were recorded. At the same time intervals, the presence of throat pain in the neutral state and during swallowing was questioned and recorded, as well as headaches. PONV^12^ (postoperative nausea and vomiting), Numeric Rank Score (NRS) for 24 h following the end of the operation (0: No nausea, vomiting, 1: Nausea, no vomiting, 2: Vomiting once, 3: Two or more episodes of vomiting ) and three different periods (NRS1: 0–2 h, NRS2: 2–6 h, NRS3: 6–24 h). 10 mg metoclopramide was given intravenously to those with an NRS value of 1 and above. None of the cases were treated for prophylaxis of nausea-vomiting. The depth of anesthesia, which suppresses the response to cuff deflating and prevents large muscle movement during tracheal extubation, was defined as “deep anesthesia”. All patients were extubated under deep anesthesia. All patients were given oxygen until they recovered from anesthesia and were observed for laryngospasm until they were discharged from the recovery unit. Although maneuvers to relieve soft tissue obstructions were performed, airway obstructions with SpO_2_ < 85% were considered as laryngospasm and were treated in accordance with the specified protocol; 1—Face mask and positive pressure ventilation with 100% O_2_, if symptoms persist; 2—IV administration of 1 mg/kg lidocaine, if symptoms still persist; 3—IV application of 1 mg/kg succinylcholine and performing tracheal intubation.

### Statistical analysis

All the analysis were carried out by mean of Windows SPSS software (IBM Corp. Released 2013. IBM SPSS Statistics for Windows, version 22.0. Armonk, NY: IBM Corp). Appropriateness of variables was investigated with visual and analytical methods. Descriptive statistical data were presented as mean, standard deviation, numbers, and percentage. The *t*-test was used when the normal distribution characteristics were compared in the comparison of the averages between the groups, and the Mann–Whitney *U* test and the Kruskal Wallis test were used in the other case. In the comparison of the ratios between the groups, the square test; Spearman or Pearson correlation tests were used according to the appropriateness of the distribution for the correlation analysis. Correlation strength was classified as follows: low, *r* ≤ 0.4; moderate, *r* = 0.4–0.5; and high, *r* ≥ 0.5 (23). The data were evaluated at a 95% confidence interval. A value of *p* < 0.05 was accepted as statistically significant.

## Results

This study was conducted with total 80 patients divided into 2 groups of 40 people. There was no statistically significant difference between the groups in terms of hemodynamic parameters (MBP, HR, ASA) (*p* > 0.05), (Table 1). The operation and surgical times of the groups were similar (*p* > 0.05).

**Table 1:** Comparison of Group S and Group C based on demographic parameters, hemodynamic parameters, and surgical interventions characteristics.

Variable	Group S (n = 40)	Group C (n = 40)	*p*-Value^b^
Age, mean ± SD years^a^	30.87 ± 7.13	31.72 ± 8.02	0.64
Gender (male/female)	19/21	18/22	0.67
BMI, mean ± SD, kg/m^2^^a^	26.04 ± 3.92	26.33 ± 4.82	0.34
ASA-PS class			0.65
Class I	24 (60%)	25(62.5%)	
Class II	16 (40%)	15 (37.5%)	
Time of surgery, mean ± SD^a^	140.35 ± 10.61	139.57 ± 9.61	0.49
Time of anesthesia, mean ± SD^a^	160.57 ± 9.24	159.10 ± 8.72	0.89
MBP, mean ± SD (mmHg)^a^	84.13 ± 10.42	85.66 ± 10.79	0.60
HR, mean ± SD (beat/min)^a^	77.56 ± 9.65	78.63 ± 13.83	0.71

ASA-PS, American Society of Anesthesiologist Physical Status; SD, standard deviation; Group S, sphenopalatine ganglion blockade; Group C, control group; MBP, mean blood pressure; HR, heart rate.

a Values are expressed as mean (SD).

b Independent *t*-test.

All VAS values were statistically lower in Group S than in Group C, as shown in Table 2 (*p* < 0.05). While sore throat in the normal position, sore throat on swallowing and headache were detected in 5, 11 and 12 cases, respectively, in Group S, these rates were found in 10, 17, and 19 cases, respectively, in Group C and were statistically significant (*p* < 0.05) (Table 2). In Group S, the need for analgesic medication was found in 5 cases between 0–2 h, whereas in Group C, this rate was found in 17 cases, and it was statistically significant (*p* < 0.05) (Table 2). In Group S, the need for analgesic medication was found in 9 and 12 cases between 2–24 h and in total, respectively, while in Group C, this rate was found in 13 and 23 cases. Although the rates were lower, the difference was not statistically significant (*p* > 0.05 (Table 2).

**Table 2:** Comparison of group S and group C based on VAS (visual pain scale) scores, sore throat, headache and analgesic requirement. The data is given as average ± standard deviation (ort ± ss).

Parameters	Group S (n = 40)	Group C (n = 40)	*p*-Value^b^
t1^a^	1.45 ± 0.78	4.95 ± 1.43	0.035
t2^a^	1.65 ± 0.73	5.37 ± 1.00	0.049
t3^a^	2.90 ± 1.10	5.87 ± 1.82	<0.001
t4^a^	2.02 ± 0.76	3.32 ± 1.11	0.011
Sore throat (normal position)	5 (12.5%)	10 (25%)	0.004
Sore throat (swallowing)	11 (27.5%)	17 (42.5%)	0.01
Headache	12 (30%)	19 (47.5%)	0.01
Analgesic requirement (0–2 h)	5 (12.5%)	17 (42.5%)	<0.001
Analgesic requirement (2–24 h)	9 (22.5%)	13 (32.5%)	0.14
Analgesic requirement (total)	14 (35%)	30 (75%)	0.058

t1, Derlenme unitesinde; t2, Postop. 2 h; t3, Postop. 6 h; t4, Postop 24 h; VAS, visual analog scale; Group S, sphenopalatine ganglion blockade; Group C, control group.

a Values are expressed as mean (SD).

b Independent *t*-test.

Postoperative nausea-vomiting was observed in 6 cases in both groups in the first 2 h. It was observed in 3 cases in Group S and 5 cases in Group C between hours 6–24 in the study. While nausea-vomiting was not observed in any patient in Group S between 6–24 h, 2 patients in Group C had nausea-vomiting. At the end of the postoperative 24th hour, nausea-vomiting was observed in 9 patients in Group S and 13 patients in Group C, but these differences were not statistically significant (*p* > 0.05) (Table 3). While laryngospasm was observed in 2 cases in Group C, laryngospasm was not observed in any case in Group S, and the difference between them was not statistically significant (*p* > 0.05) (Table 3).

**Table 3:** Numic Rank Score of Groups (NRS) and laryngospasm frequency.

Parameters	Group S (n = 40)	Group C (n = 40)	*p*^b^
NRS1 (0/1/2)	34/4/2 (85/10/5%)	34/3/3 (85/7.5/7.5%)	*p* > 0.05
NRS2 (0/1/2)	37/3 (92.5/7.5%)	35/4/1 (87.5/10/2.5%)	*p* > 0.05
NRS3 (0/1)	40/0 (100/0%)	38/1/1 (95/2.5/2.5%)	*p* > 0.05
NRS total (0/1/2)	31/7/2 (77.5/17.5/5%)	27/8/5 (67.5/20/12.5%)	*p* > 0.05
Laryngospasm	0 (0%)	3 (7.5%)	*p* > 0.05

NRS, Numerical Rank Score (0: no nausea, no vomiting, 1: nausea, no vomiting, 2: vomiting once, 3: two or more episodes of vomiting) in three separate periods (NRS1: 0–2 h, NRS2: 2–6 h, NRS3: 6–24 h); Group S: sphenopalatine ganglion blockade; Group C, control group.

## Discussion

Septorhinoplasty surgery is an operation that includes both septum surgery and rhinoplasty in order to increase the quality of life of patients, accompanied by postoperative pain and nausea, and a high consumption of analgesics.

The VAS (visual analog scale) values of the patients in the sphenopalatine ganglion block (SPGB) group, which was the primary aim of our study, were lower and statistically significant in the first 24 h compared to the control group (*p* < 0.001).

We found that patients in Group S needed less analgesics in the first 2 h than patients in Group C (*p* < 0.05). Sore throat in normal position, sore throat during swallowing and headache were lower in Group S compared to Group C and were statistically significant (*p* < 0.05). In our study, there was no difference between the groups in terms of analgesic drug need in 2–24 h, Numerical Rank Score (NRS) and laryngospasm incidence.

SPG is one of the four parasympathetic ganglia in the cranium and can also be named Meckel’s ganglion, sphenomaxillary ganglion and nasal ganglion.^13^ SPG carries only preganglionic parasympathetic axons. Both postganglionic and somatic sensory afferent branches pass near the SPG.^10^ Thus, these nerves can also be involved during SPGB. Considering this anatomical structure, we think that the beneficial effects obtained with SPGB in our study also contributed to the blocking of nerves that course adjacent to the ganglion. SPGB is a viable anesthesia and analgesia technique for Septorhinoplasty due to its advantages of providing analgesia and a bloodless surgical field.^14^ SPGB can be done in 3 different ways; 1. Lateral or zygomatic pathway, 2. Anterior or nasal pathway, 3. Inferior or palatine pathway.^15^ In our study, the nasal route was used due to the surgeon's preference and ease of application. Although bleeding at the needle insertion site can be observed rarely in nasal technique due to palatine artery trauma, this complication was not observed in any of our study patients. Pain management is very difficult because of the postoperative complications that reduce the quality of life of the patients. This postoperative pain control has been widely studied. In the study of Riley et al. they found that postoperative pain occurs mostly in the first 24 h.^16^ In our study, we evaluated the first 24 h and found that the mean pain was lower, and the pain levels were lower in patients who underwent SPGB.

Sclafani et al. evaluated the pain levels and opioid needs of patients after septorhinoplasty operations in their study. They found that NSAIDs, gabapentin, alpha-agonists, and local anesthetics were as effective as opioid alternatives in terms of perioperative analgesia after septorhinoplasty operations.^17^ In our study, we found that SPGB applied peroperatively reduced postoperative pain scores and was an effective option by providing minimum analgesic consumption. Besides PONV (post-operative nausea and vomiting) are common complications, aspiration pneumonia is an unpleasant complication that increases intracranial pressure, increases bleeding, electrolyte imbalance, and prolongs hospital stay. In the study conducted by Brown et al., the data of 3962 patients including all kinds of otolaryngology operations were examined and they found that 30% of hospitalizations were due to vomiting.^18^ Abubaker et al., in their study, found that SPGB with lidocaine at the end of endoscopic sinus surgery was a safe, noninvasive effective method in reducing early PONV.^19^ In our study, long-acting bupivacaine was preferred instead of lidocaine, and although less nausea and vomiting was observed in the SPGB applied group, it was not statistically significant.

There were some limitations in our study. First, we evaluated pain intensity only for the first 24 h. We could have evaluated it for a longer time (48–72 h). Second, some patients had pain (if VAS ≥ 5 points in the first 4 h after the operation), 1 mg/kg diclofenac sodium was used. We think that these patients will have less pain in the coming hours, which is an advantage. Despite taking NSAIDs, these patients still had higher VAS values in the ongoing process compared to the group in which SPG blockade was applied. The same situation can occur with PONV. The other one may seem disadvantageous as we will artificially prolong the operation time with the SPGB procedure, so we have not considered it.

## Conclusion

Our findings show that SPGB is very effective in postoperative pain control in Septorhinoplasty surgery and provides less exposure to drug side effects by reducing postoperative analgesia consumption. Further studies are needed for laryngospasm, nausea and vomiting. As a result, we showed that SPGB is a cost-effective, easy-to-apply, less side-effect and pain-reducing method, as observed in previous clinical studies.

## Ethics approval

This study was reviewed and approved by the institutional review board at the Health Sciences University Diyarbakir Gazi Yasargil Training and Education hospital, ID: 66, 2022. Written informed consent was obtained from all patients.

## Funding

The study was funded by departmental resources.

## Conflicts of interest

The authors declare no conflicts of interest.

## CRediT authorship contribution statement

**Erhan Gökçek:** Conceptualization, Validation, Data curation, Supervision, Funding acquisition. **Gunay Kozan:** Conceptualization, Methodology, Software, Validation, Formal analysis, Investigation, Resources, Data curation, Writing – original draft, Writing – review & editing, Visualization, Project administration.
